# Supplementary material for: How Single Amino Acid Substitutions Can Disrupt a Protein Hetero-Dimer Interface: Computational and Experimental Studies of the LigAB Dioxygenase from Sphingobium sp. Strain SYK-6
Source: Int J Mol Sci. 2023 Mar 28;24(7):6319. doi: 10.3390/ijms24076319 (PMC10094722; doi:10.3390/ijms24076319)
Supplement: Supplementary file 1 [file ijms-24-06319-s001.zip › ijms-2207450-supplementary.pdf]

Supplemental figures for **How Single Amino Acid Substitutions can Disrupt a Protein Hetero-Dimer Interface: Computational and Experimental Studies of the LigAB Dioxygenase from *Sphingobium* sp. Strain SYK-6**

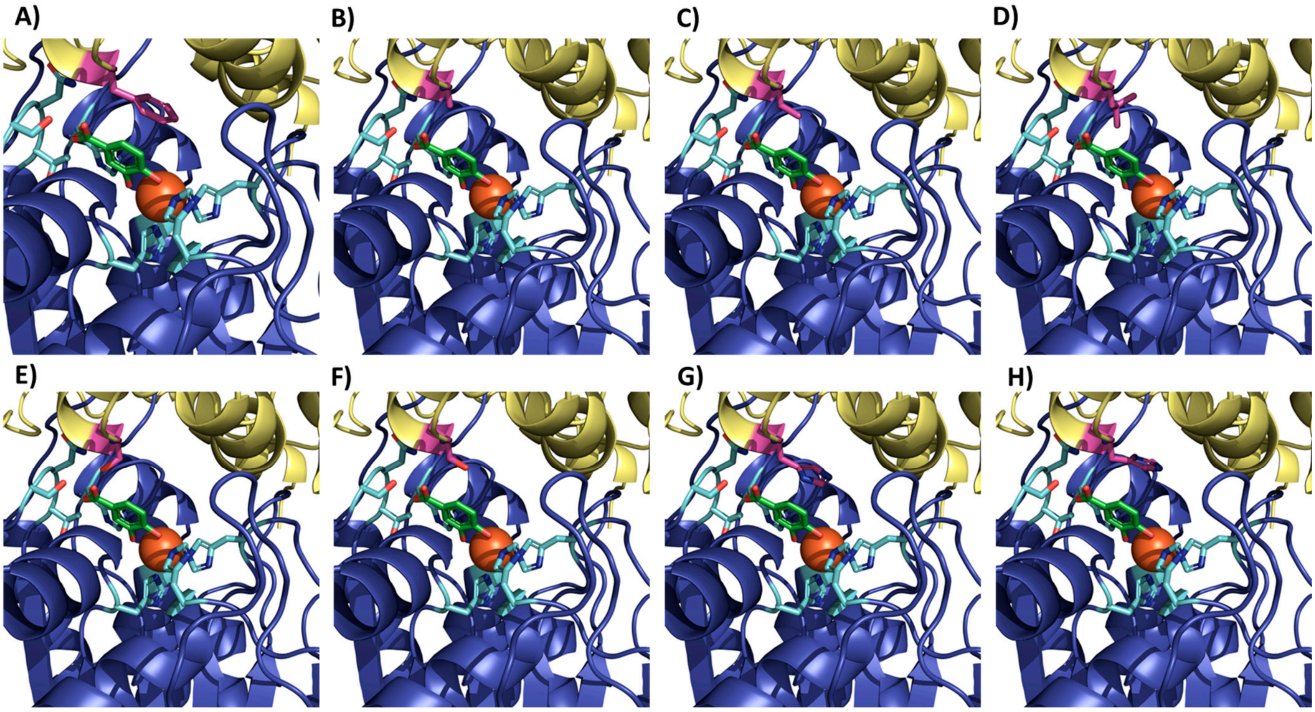

Figure S1: Active site of the LigAB complex with mutations at the 103α residue. A close-up of the active site with the active site residues in yellow and F103α residue in pink for **(A)** wild-type and corresponding mutations **(B)** alanine, **(C)** valine, **(D)** leucine, **(E)** serine, **(F)** threonine, and histidine protonated at the **(G)** δ or **(H)** ε nitrogen.

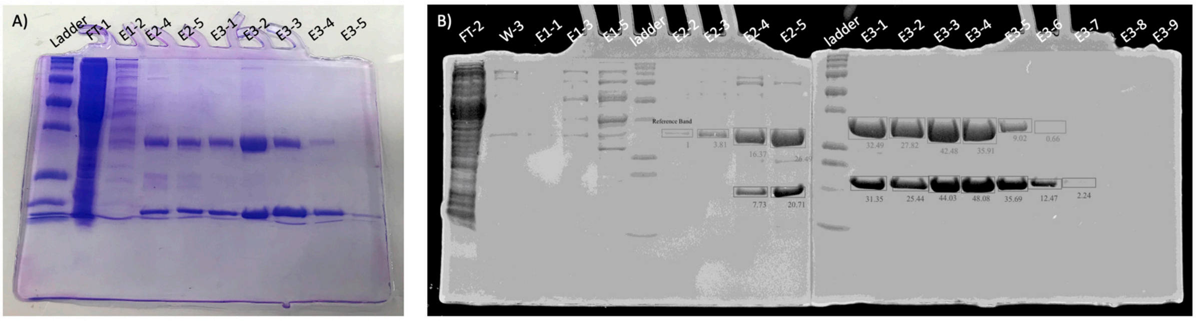

Figure S2: Wild-type LigAB purifications visualized by SDS-PAGE. A) A Representative SDS-PAGE gel of anaerobically purified LigAB and **B)** a SDS-PAGE gel of aerobically purified LigAB used in this study. Both gels were stained with Coomasie Blue.

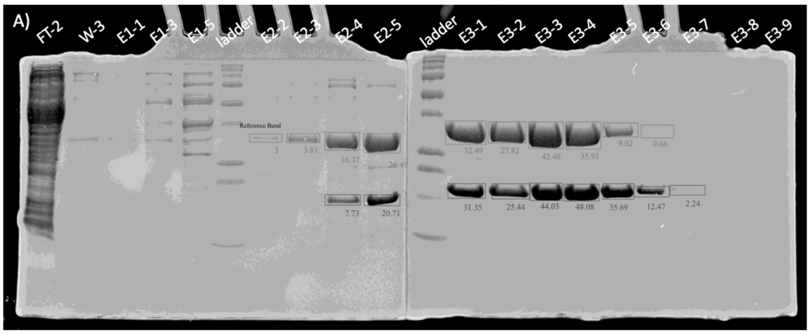

B)

wt	α	β
E3-1	49.1%	50.9%
E3-2	47.8%	52.2%
E3-3	50.9%	49.1%
E3-4	57.2%	42.8%
E3-5	79.8%	20.2%

Figure S3: Quantified percentages of α and β subunits from the wild-type LigAB purification. A) The SDS-PAGE gel from the purification of wild-type LigAB stained using Coomasie Blue. The reference band is marked and the weights of each of the bands are written on the gel. B) A table with the calculated percentages in fractions of LigA and LigB that are collected for concentration and used for protein characterization for wild-type LigAB.

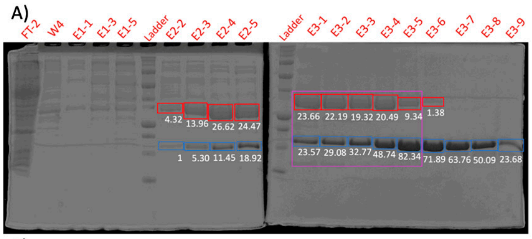

B)

F103A	α	β
E3-1	49.0%	50.1%
E3-2	56.7%	43.3%
E3-3	62.9%	37.1%
E3-4	70.4%	29.6%
E3-5	89.8%	10.2%

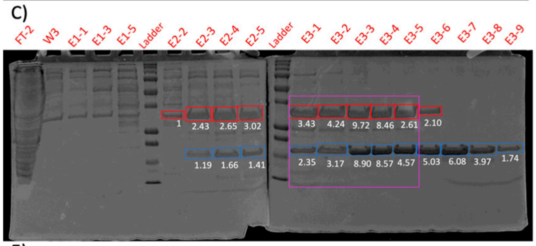

D)

F103V	α	β
E3-1	40.7%	59.3%
E3-2	42.8%	57.2%
E3-3	47.8%	52.2%
E3-4	50.3%	49.7%
E3-5	63.6%	36.4%

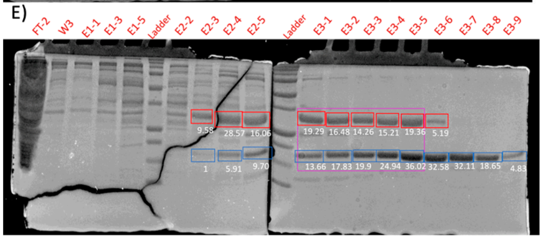

F)

F103L	α	β
E3-1	41.5%	58.5%
E3-2	52.0%	48.0%
E3-3	58.3%	41.3%
E3-4	62.1%	37.9%
E3-5	65.0%	35.0%

Figure S4: Quantified percentages of α and β subunits from the nonpolar mutant purifications. : The SDS-PAGE gels stained with Coomasie Blue and tables with the percentages of LigA and LigB in fractions

that would be taken for further concentrating for further studies of nonpolar mutants: A) and B) F103A, C) and D) F103V, E) and F) F103L.

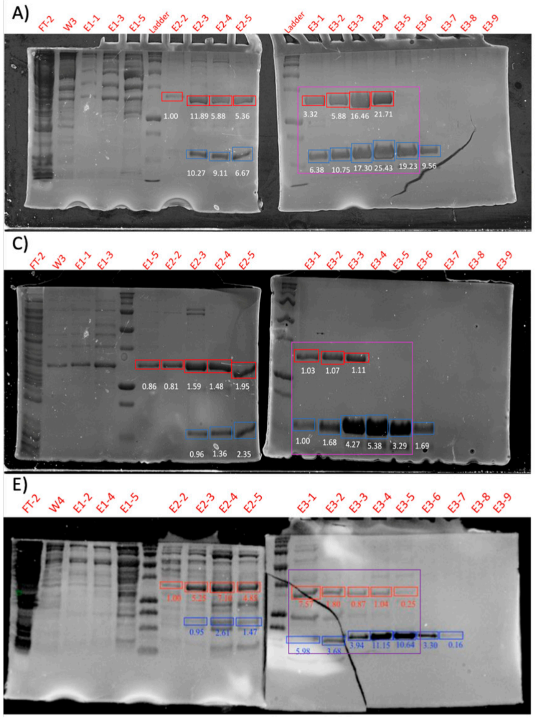

B)

F103S	α	β
E3-1	65.8%	34.2%
E3-2	64.6%	35.4%
E3-3	51.2%	48.8%
E3-4	52.9%	46.1%
E3-5	100.0%	0.0%

D)

F103H	α	β
E3-1	49.3%	50.7%
E3-2	61.1%	38.9%
E3-3	79.4%	20.6%
E3-4	100.0%	0.0%
E3-5	100.0%	0.0%

F)

F103T	α	β
E3-1	44.2%	55.9%
E3-2	67.2%	32.8%
E3-3	81.9%	18.1%
E3-4	91.5%	8.5%
E3-5	97.7%	2.3%

Figure S5: Quantified percentages of α and β subunits from the polar mutant purifications. : The SDS-PAGE gels stained with Coomasie Blue and tables with the percentages of LigA and LigB in fractions that would be taken for further concentrating for further studies of polar mutants: A) and B) F103S, C) and D) F103H, E) and F) F103T.

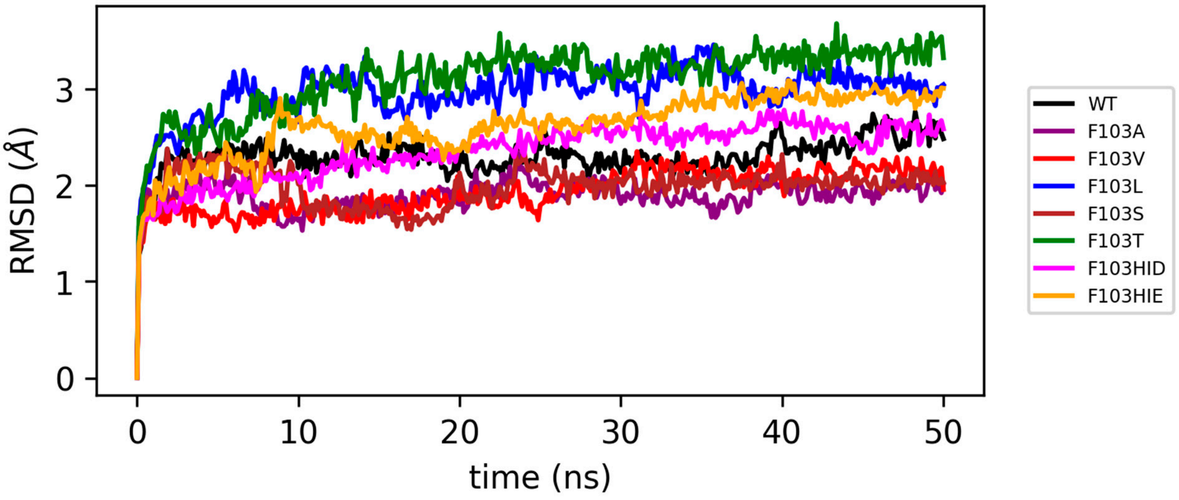

Figure S6: Backbone root mean square deviation of wild-type and F103α mutants.. : RMSD of LigAB heterodimer relative to the equilibrated structure as a function of time. All variants equilibrate to under approximately 3.5 Å.

A) **B)**

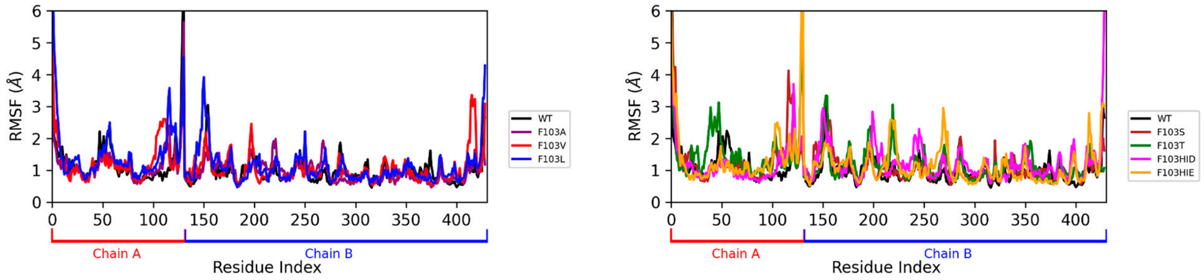

Figure S7: Root mean square fluctuations of wild-type and F103α mutants.. : The average C_α root mean square fluctuation of the LigAB heterodimer. Residues in the A and B chain are labeled on the x axis. **(A)** Most differences in fluctuations occur adjacent to the mutation (residues >100) in chain A for variants alanine, valine, and leucine. **(B)** Whereas, fluctuations in chain B mainly occur in the polar residue variants including serine, threonine, and histidines.

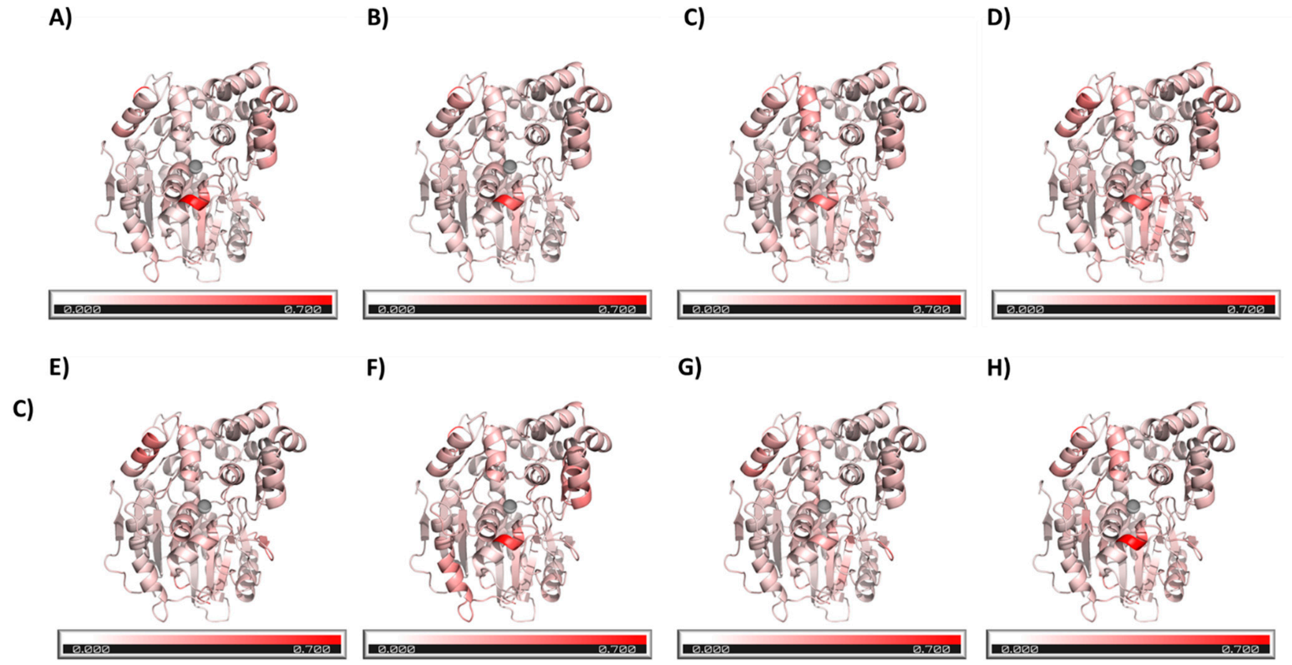

Figure S8: Calculated RMSF mapped to crystal structures of wild-type and F103α mutants.. The average C_α root mean square fluctuation of the LigAB heterodimer mapped onto the crystal structure for **(A)** wildtype, **(B)** alanine, **(C)** valine, **(D)** leucine, **(E)** serine, **(F)** threonine, and histidine protonated at the **(G)** δ or **(H)** ε nitrogen.

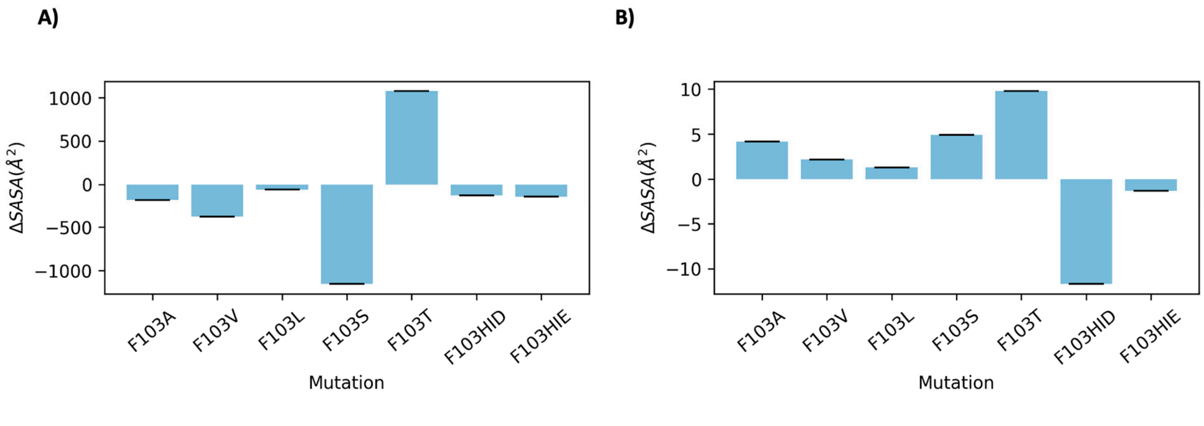

Figure S9: Solvent accessible surface area of F103α mutants relative to wild-type (ΔSASA) (A) for the dimer as a whole and **(B)** for the difference for the individual mutated residues.
